# Downregulation of kinetochore-associated 1 gene increases lagging chromosomes and contributes to chromosomal instability in gastric cancer cells

**DOI:** 10.3892/mi.2025.258

**Published:** 2025-08-04

**Authors:** Daiki Ohsaki, Kazuki Kanayama

**Affiliations:** 1 Graduate School of Health Science, Suzuka University of Medical Science, Suzuka, Mie 510-0293, Japan; 2 Department of Medical Technology, Suzuka University of Medical Science, Suzuka, Mie 510-0293, Japan

**Keywords:** gastric cancer, receptor tyrosine kinase, chromosomal instability, lagging chromosome, kinetochore-associated 1 gene

## Abstract

Gastric cancer (GC) is classified into four molecular subtypes according to the Epstein-Barr virus-positive status, microsatellite instability, genomic stability and chromosomal instability (CIN). The CIN subtype is characterized by a high frequency of gene amplifications in receptor tyrosine kinases (RTKs) and a poor prognosis. In addition, the CIN subtype often exhibits intratumoral heterogeneity and indicates insensitivity to targeted drugs. Elucidating the molecular mechanisms of CIN in GC is therapeutically crucial; however, the molecular mechanisms involved are not yet fully understood. The kinetochore-associated 1 (*KNTC1*) gene encodes kinetochore-associated protein 1 (KNTC1), a major component of the outer kinetochore. The downregulation of *KNTC1* causes a high frequency of lagging chromosomes and consequent aneuploidy and CIN in *Drosophila* and *Caenorhabditis elegans*. However, the association between *KNTC1* and CIN in GC has not yet been clarified. Therefore, the present study investigated the role of *KNTC1* in GC CIN. It was found that GC cell lines with a high frequency of lagging chromosomes had a low *KNTC1* mRNA expression. Notably, *KNTC1* knockdown increased the frequency of lagging chromosomes in GC cell lines. In particular, GC cell lines with the amplification of RTK genes exhibited a significant increase in the frequency of lagging chromosomes. On the whole, the findings of the present study suggest that the suppression of *KNTC1* expression may contribute to CIN in GC and may be involved in the generation of intratumoral genetic heterogeneity in GC.

## Introduction

In 2020, ~770,000 patients succumbed due to gastric cancer (GC), rendering GC the fourth most common cause of cancer-related mortality worldwide, surpassed only by lung, colorectal and liver cancers (1). As GC exhibits histological and genetic heterogeneity, the development of biomarker-based molecularly targeted therapeutics has lagged behind that of other cancer types (2). However, The Cancer Genome Atlas research program has succeeded in characterizing GC at the genomic level, yielding a system for classifying GC into four molecular subtypes according to the Epstein-Barr virus-positive status, microsatellite instability, genomic stability and chromosomal instability (CIN), with the aim of simplifying the treatment and diagnosis of GC (2,3).

The CIN subtype accounts for ~50% of GC cases and is characterized by the amplification of receptor tyrosine kinase (RTK) genes, a high frequency of *TP53* mutations (in 70% of cases) and CIN, which is indicated by a high frequency of aneuploidy (4,5). In addition, the CIN subtype is known to exhibit intratumoral heterogeneity, which is involved in tumor relapse owing to the acquisition of cellular insensitivity to targeted drugs (6-8). Therefore, elucidating the molecular mechanisms of CIN in GC is of utmost therapeutic importance. However, these mechanisms are not yet fully understood.

The kinetochore-associated 1 (*KNTC1*) gene encodes kinetochore-associated protein 1 (KNTC1), a protein component of the outer kinetochore that is essential for the association of chromosomes and spindle microtubules. KNTC1 forms a complex with ZW10 and ZWILCH during mitosis. This complex, which is known as the RZZ complex, is involved in the activation of the spindle assembly checkpoint (SAC), the kinetochore-dependent recruitment of the Mad1/Mad3 and dynein/dynactin complex and the formation of the kinetochore corona in the outermost layer of the kinetochore (9-14). In particular, the activation of the SAC delays anaphase when there is a lack of proper connection between kinetochores and spindle microtubules, allowing for homogenous chromosome segregation (15,16). The depletion or loss of function of various kinetochore proteins, including KNTC1, has been reported to cause lagging chromosomes, resulting in abnormal chromosome segregation and subsequent aneuploidy and CIN in *Drosophila* and *Caenorhabditis elegans (*16-18). However, the role of the *KNTC1* gene in GC is poorly understood.

It was hypothesized that the abnormal function of *KNTC1* may be associated with the mechanism of CIN in GC. Therefore, the present study investigated the role of *KNTC1* in GC CIN.

## Materials and methods

### Cell lines and culture conditions

The human GC cell lines, NCI-N87 (exhibiting human epidermal growth factor receptor type 2 gene amplification; cat. no. CRL-5822, American Type Culture Collection), KATOIII [exhibiting fibroblast growth factor receptor type 2 gene amplification; cat. no. JCRB0611, Japan Collection of Research Bioresources (JCRB) Cell Bank] and MKN74 (without amplification of RTK genes; cat. no. JCRB0255, JCRB), and the human normal fibroblast cell line, TIG-1-20 (cat. no. JCRB0501, JCRB), were used in the present study. The cells were cultured in RPMI-1640 medium (cat. no. 30264-56; Nacalai Tesque, Inc.) containing a 10% fetal bovine serum and 0.5% penicillin and streptomycin mixture (cat. no. 09367-34; Nacalai Tesque, Inc.) at 37˚C in an atmosphere containing 5% CO_2_. The cell cultures were grown in a CO_2_ incubator (cat. no. MHE-S1301A2-PJ; PHC Holdings Corporation).

### Measurement of the frequency of lagging chromosomes

**Figure 1:**
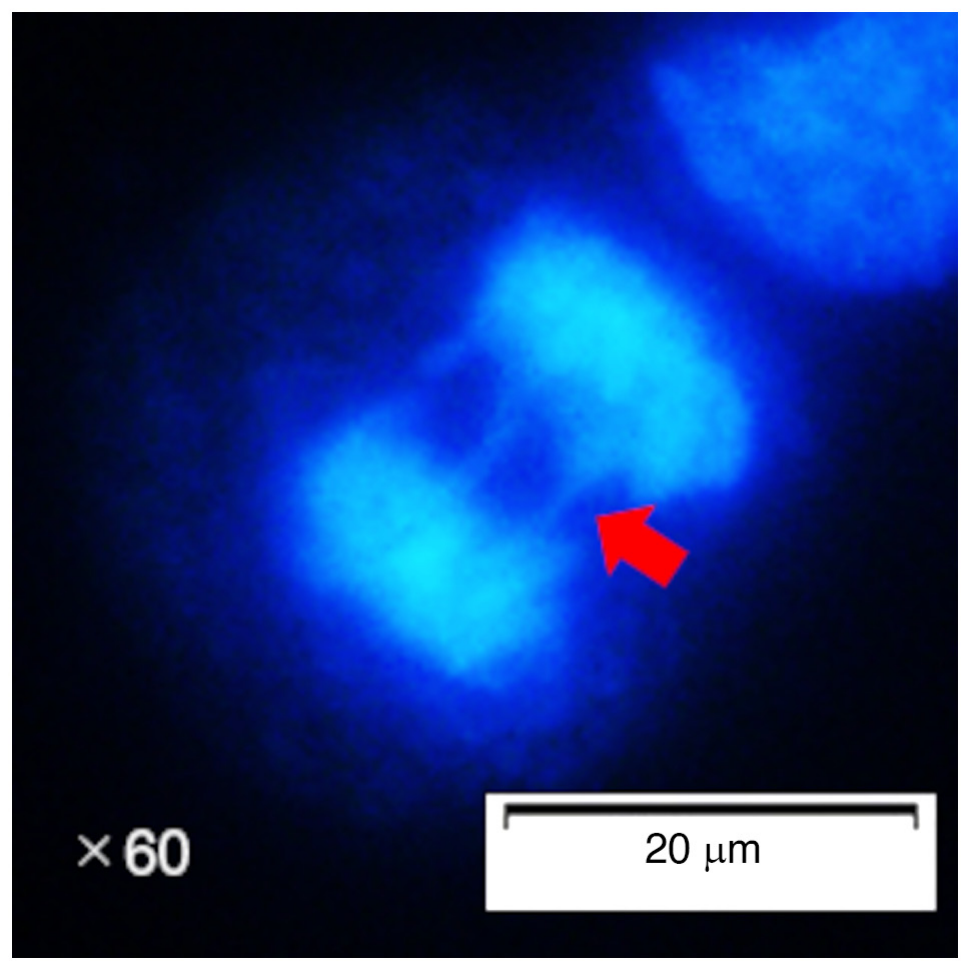
Representative image of lagging chromosomes in NCI-N87 cells. Chromosomes remained on the metaphase plate and formed bridges in anaphase (red arrow).

The NCI-N87, KATOIII, MKN74 and TIG-1-20 cells were seeded into 4-well culture slides (cat. no. 192-004; Watson Bio Lab) and cultured for 24 h at 37˚C. After 24 h, the cells were transfected with small interfering RNA (siRNA) targeting *KNTC1*, as described below. Following transfection, the cells are incubated for an additional 3 days and fixed for 20 min at room temperature with 4% paraformaldehyde (cat. no. 006775-1L; Bioenno Tech, LLC). The slides were washed twice for 5 min each with phosphate-buffered saline (cat. no. 73111; Kanto Chemical Co.) and sealed using coverslips and VECTASHIELD Vibrance Antifade Mounting Medium with DAPI (cat. no. H-1800; Vector Laboratories, Inc.). A total of 50 cells in anaphase, defined according to visible sister chromatid separation, were then observed using a fluorescence microscope (BX53F; Olympus Corporation). Among these 50 cells, the number of lagging chromosomes was counted (Figs. 1 and S1), and the percentage was calculated.

### Reverse transcriptionquantitative polymerase chain reaction (RTqPCR)

Total RNA was extracted from the NCI-N87, KATOIII, MKN74 and TIG-1-20 cells using an RNeasy mini kit (cat. no. 74104; Qiagen, Inc.). cDNA synthesis was performed using reverse transcription with Superscript Ⅳ VILO Master Mix with ezDNase (cat. no. 11766050; Invitrogen; Thermo Fisher Scientific, Inc.). cDNA synthesis reaction was performed at 25˚C for 10 min, 50˚C for 10 min, and 85˚C for 5 min. *KNTC1* (Hs00938554_m1) and *GAPDH* (No. 1902206) primers were obtained from Thermo Fisher Scientific, Inc. (primer sequence information not available). qPCR was performed using TaqMan Fast Advanced Master Mix (cat. no. 4444556; Applied Biosystems; Thermo Fisher Scientific, Inc.) using the following reaction conditions: An initial denaturation at 95˚C for 20 sec, then 40 cycles at 95˚C for 3 sec and 60˚C for 30 sec. mRNA expression was analyzed using the 2^-ΔΔCq^ calculation (19).

### Western blot analysis

The NCI-N87, KATOIII, MKN74 and TIG-1-20 cells were lysed in RIPA buffer (cat. no. 08714-04; Nacalai Tesque, Inc.). The cell lysates were then treated using ultrasound and centrifuged at 20,630 x g for 10 min at room temperature. The lysates were mixed with sample buffer (cat. no. 30566-22; Nacalai Tesque, Inc.) with 2-mercapto ethanol and incubated at 100˚C for 3 min. Lysates containing 5-7 µg protein were separated by 10% sodium dodecyl sulfate-polyacrylamide gel electrophoresis using Mini Trans-Blot^®^ Cell (cat. no. 153BR78145; Bio-Rad Laboratories, Inc.). The separated proteins were then transferred from the gel to polyvinylidene fluoride membranes (cat. no. 1704272; Bio-Rad Laboratories, Inc.) using the Trans-Blot^®^Turbo^TM^ System (cat. no. 690BR009070; Bio-Rad Laboratories, Inc.). The membranes were blocked for 5 min at room temperature with Every Blot blocking buffer (cat. no. 12010020; Bio-Rad Laboratories, Inc.) and incubated with anti-ZW10 (1:1,000; cat. no. 24561-1-AP, Proteintech, Inc.) and anti-α-tubulin (1:5,000; cat. no. 3873, clone DM1A, Cell Signaling Technology, Inc.) antibodies overnight at 4˚C. Following incubation, the membranes were washed in Tris-buffered saline containing 0.1% Tween-20 (TBS-T). The membranes were then incubated with appropriate secondary antibodies (1:5,000; anti-mouse IgG, cat. no. 7076, Cell Signaling Technology, Inc. and 1:5,000; anti-rabbit IgG, cat. no. 7074, Cell Signaling Technology, Inc.) for 1 h at room temperature and washed with TBS-T. Protein bands were detected using a ChemiDoc MP Imaging System (Bio-Rad Laboratories, Inc.). Quantitative analysis was performed using ImageLab software version 4.1 (Bio-Rad Laboratories, Inc.).

### siRNA-mediated knockdown of KNTC1

The sequence (GGAAUGAUAUUGAGCUGCUAACAAA) of human *KNTC1* siRNA was designed from Thermo Fisher Scientific, Inc. (cat. no. HSS114610). *KNTC1* siRNA or negative control (cat. no. 12935-300; Invitrogen; Thermo Fisher Scientific, Inc.) was combined with Lipofectamine RNAiMAX (cat. no. 13778-030; Invitrogen; Thermo Fisher Scientific, Inc.) and incubated for 15 min at room temperature. The NCI-N87, KATOIII, MKN74 and TIG-1-20 cells were transfected with the *KNTC1* siRNA or negative control-Lipofectamine RNAiMAX complexes and incubated at 37˚C for 3 days. At 3 days following the addition of the *KNTC1* siRNA or negative control-Lipofectamine RNAiMAX complexes, the cells were harvested and processed for RT-qPCR and western blot analysis, and for the analysis of the frequency of lagging chromosomes.

### Statistical analysis

All the statistical analyses were performed using Statcel 4 (https://oms-publ.main.jp/main/4steps4-hyo1/). The one-way ANOVA and Tukey-Kramer test were performed to assess differences in frequency of lagging chromosomes and *KNTC1* mRNA expression. The Student's t test was used to assess the efficiency of *KNTC1* knockdown and the frequency of lagging chromosomes and ZW10 protein expression following *KNTC1* knockdown. Data are presented as the mean ± standard deviation. P-values #x003C;0.05 were considered to indicate statistically significant differences.

## Results

### Frequency of lagging chromosomes and expression levels of KNTC1 mRNA

**Figure 2:**
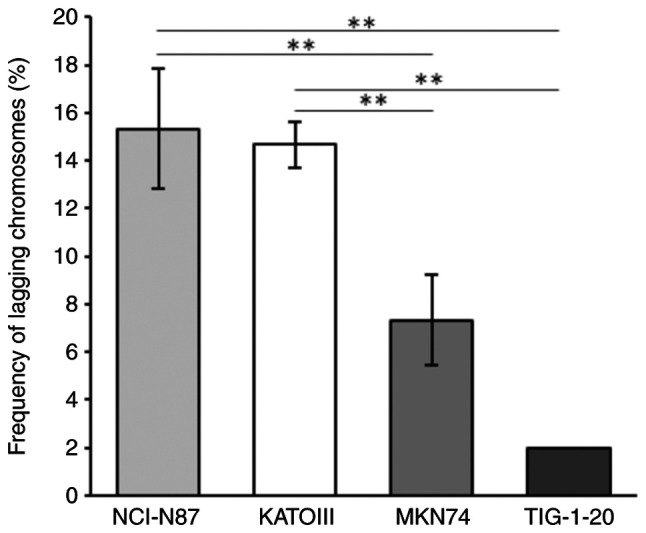
Frequency of lagging chromosomes in the NCI-N87, KATOIII, MKN74 and TIG-1-20 cells. The frequency of lagging chromosomes was significantly higher in the NCI-N87 and KATOIII cells than in the MKN74 cells. All data were analyzed using one-way ANOVA and Tukey-Kramer test. ^**^P#x003C;0.01.

To investigate the CIN status in GC cells, the frequency of lagging chromosomes was analyzed. The frequency of lagging chromosomes was significantly higher in the NCI-N87 and KATOIII cells, which exhibited the amplification of RTK genes, than in the MKN74 cells, which did not exhibit the amplification of RTK genes (Fig. 2). Only a small number of lagging chromosomes were observed in the TIG-1-20 cells.

**Figure 3:**
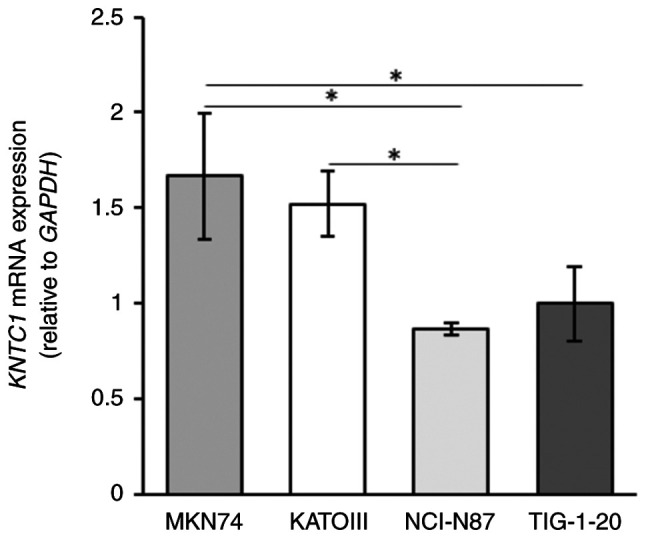
mRNA expression levels of *KNTC1* in the NCI-N87, KATOIII, MKN74 and TIG-1-20 cells, as measured using reverse transcription-quantitative PCR. *KNTC1* mRNA expression was higher in the MKN74 cells than in the NCI-N87 and KATOIII cells and was inversely associated with the frequency of lagging chromosomes. All data were analyzed using one-way ANOVA and Tukey-Kramer test. ^*^P#x003C;0.05. *KNTC1*, kinetochore-associated 1 gene.

The expression levels of *KNTC1* mRNA were higher in the MKN74 cells than in the NCI-N87 and KATOIII cells, and were inversely associated with the frequency of lagging chromosomes (Fig. 3). In addition, the mRNA expression level of *KNTC1* in the NCI-N87 cells was significantly lower than that in TIG-1-20 cells.

### Frequency of lagging chromosomes and expression of ZW10 following KNTC1 knockdown

**Figure 4:**
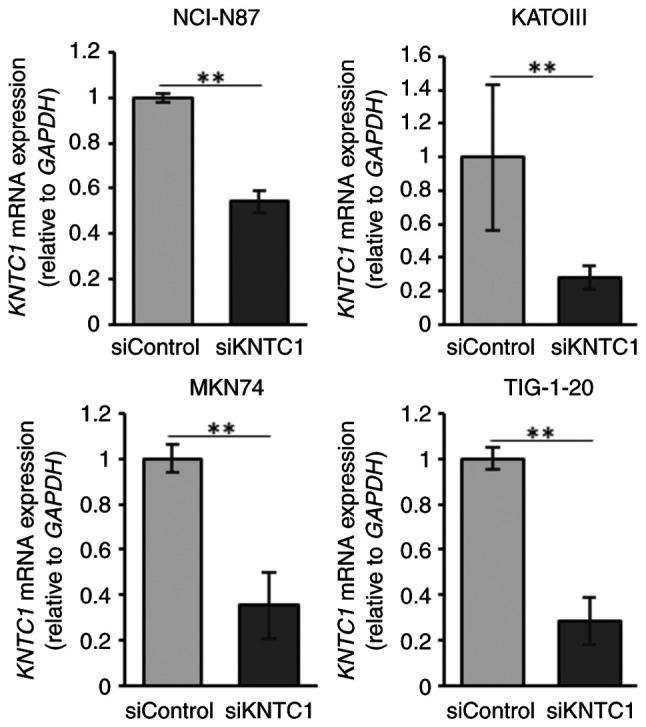
The NCI-N87, KATOIII, MKN74 and TIG-1-20 cells were transfected with siRNA targeting *KNTC1* or negative control (siControl). At 3 days following transfection, total RNA was extracted and measured using reverse transcription-quantitative PCR. *KNTC1* mRNA expression was significantly decreased following transfection with siRNA targeting *KNTC1* in all cell lines. All data were analyzed using the Student's t-test. ^**^P#x003C;0.01 vs. siControl *KNTC1*, kinetochore-associated 1 gene.

**Figure 5:**
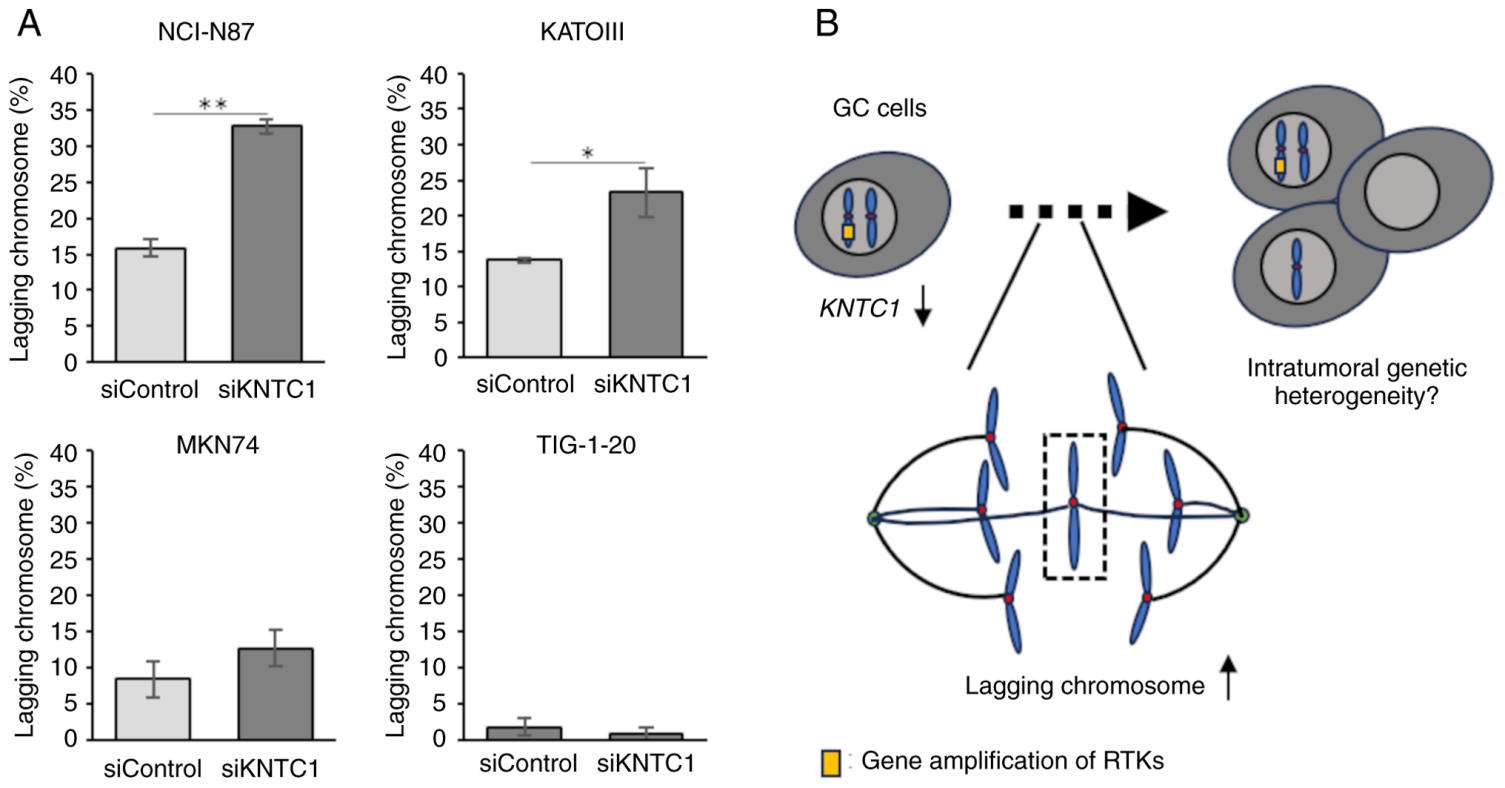
Frequencies of lagging chromosomes in NCI-N87, MKN74, TIG-1-20 and KATOIII cells transfected with siRNA targeting *KNTC1* or negative control (siControl) for 3 days. (A) The frequency of lagging chromosomes was significantly increased in the NCI-N87 and KATOIII cells transfected with siRNA targeting KNTC1. All data were analyzed using the Student's t-test. ^*^P#x003C;0.05 vs. siControl; ^**^P#x003C;0.01 vs. siControl. (B) The silencing off KNTC1 could lead to intratumoral genetic heterogeneity in GC cells with the amplification of RTK genes. *KNTC1*, kinetochore-associated 1 gene; GC, gastric cancer; RTKs, receptor tyrosine kinases.

The present study then investigated whether the knockdown of *KNTC1* increased the frequency of lagging chromosomes. The confirmation of the knockdown efficiency *KNTC1* siRNA was performed using RT-qPCR (Fig. 4). The NCI-N87, KATOIII and MKN74 cells exhibited increased frequencies of lagging chromosomes following the knockdown of *KNTC1* (Fig. 5A). These differences were statistically significant in the NCI-N87 and KATOIII cells. By contrast, no increases in lagging chromosomes were observed in the TIG-1-20 cells following the knockdown of *KNTC1*.

**Figure 6:**
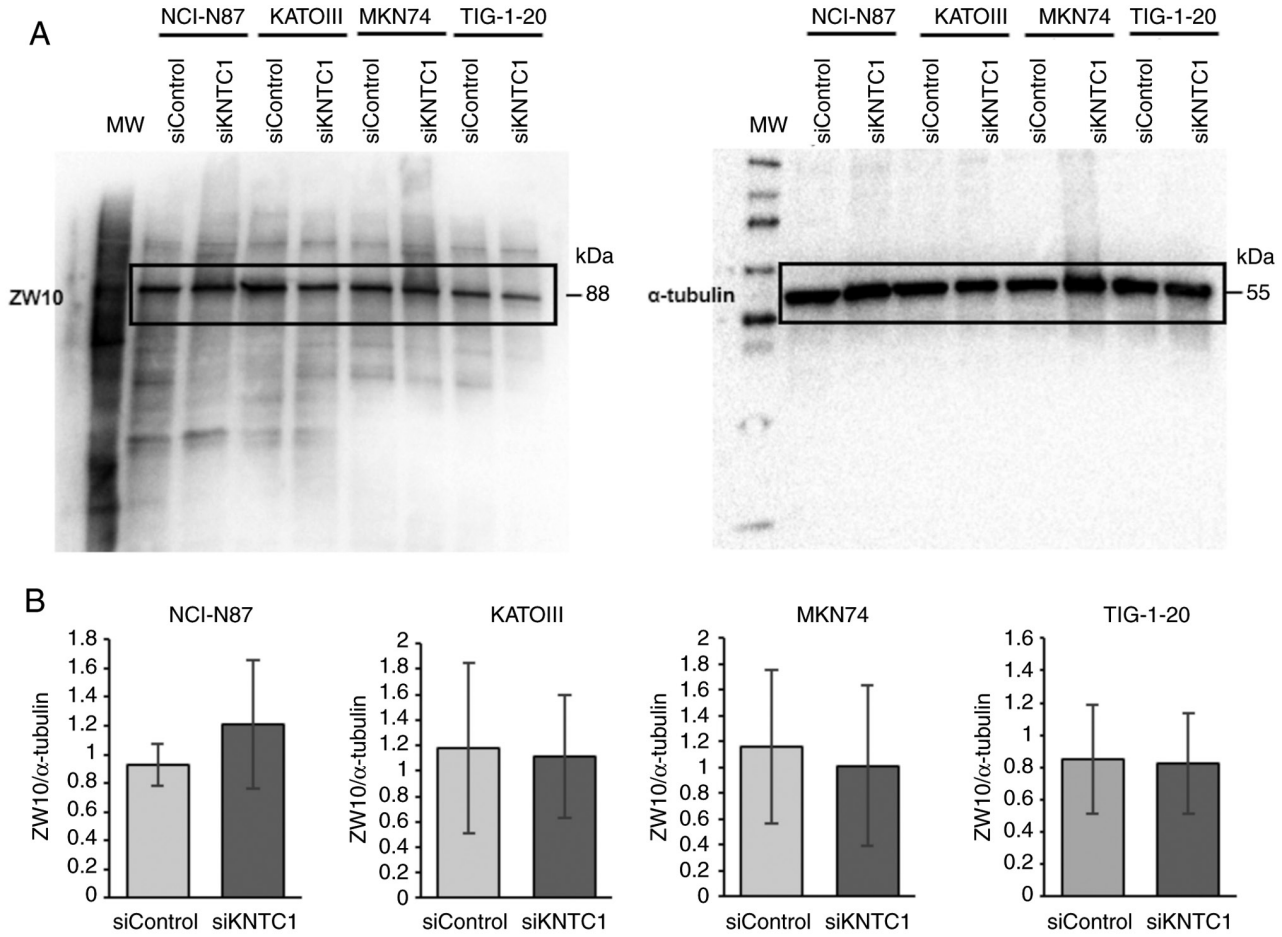
ZW10 expression in NCI-N87, KATOIII, MKN74 and TIG-1-20 cells. Cells were transfected with siRNA targeting *KNTC1* or negative control (siControl). (A) ZW10 expression was evaluated using western blot analysis, with α-tubulin as the loading control. (B) Quantitative analysis using ImageLab software. No change in ZW10 expression was observed following the knockdown of *KNTC1*. All data were analyzed using the Student's t-test. *KNTC1*, kinetochore-associated 1 gene.

In addition, to determine whether ZW10 compensated for KNTC1 function, ZW10 protein expression was investigated following *KNTC1* knockdown. However, no changes in ZW10 protein expression were observed in any of the cell lines tested (Fig. 6).

## Discussion

Deletion or loss of function of various kinetochore proteins, including KNTC1, has been reported to cause chromosome segregation abnormalities and induce aneuploidy and CIN in *Drosophila* and *C. elegans (*16-18). However, the role of *KNTC1* in GC CIN is poorly understood. In the present study, it was found that GC cells with the amplification of RTK genes, including NCI-N87 and KATOIII cells, exhibited lower mRNA expression levels of *KNTC1* and *KNTC1* expression exhibited an inverse association with the frequency of lagging chromosomes (Figs. 2 and 3). Moreover, the frequency of lagging chromosomes in the NCI-N87, KATOIII and MKN74 cells increased following the knockdown of *KNTC1* (Fig. 5A). These findings suggest that the suppression of the expression of the *KNTC1* gene may contribute to CIN in GC. In particular, suppression of the expression of *KNTC1* in GC cells exhibiting the amplification of RTK genes may enhance CIN and could lead to intratumoral genetic heterogeneity (Fig. 5B). Therefore, the restoration of normal *KNTC1* expression levels may improve patient prognosis by alleviating intratumoral genetic heterogeneity through appropriate kinetochore-microtubule attachments.

ZW10 is located in the cytoplasm and endoplasmic reticulum (ER) during the interphase and is involved in transport between the ER and Golgi apparatus (20). During mitosis, ZW10 and KNTC1 are recruited to the kinetochore, where they form the RZZ complex with ZWILCH (21). When KNTC1 is present, the RZZ complex activates the SAC and recruits dynein/dynactin during mitosis. Therefore, it was hypothesized that ZW10 may compensate for the loss of function of KNTC1. However, ZW10 protein expression levels were not altered when *KNTC1* was knocked down in all cell lines (Fig. 6). This finding is consistent with the findings of a previous study demonstrating that ZW10 protein levels were not altered by the expression of a mutant *KNTC1* gene, which severely affected the localization of ZW10 in *Drosophila* (22); this suggests that ZW10 does not compensate for the function of KNTC1. This finding also suggests that there may be no association between ZW10 and CIN.

The overexpression of the *KNTC1* gene has been observed in several types of cancer, suggesting that *KNTC1* promotes cell proliferation and viability (23-25). However, in the present study, no inhibition of cell proliferation or increase in apoptotic bodies were observed following *KNTC1* knockdown in KATOIII or MKN74 cells (Data S1 and Fig. S2), although increases in lagging chromosomes were observed. In the NCI-N87 cells, differences in cell proliferation were observed at 72 h following *KNTC1* knockdown, but no apoptosis was observed. This difference suggests that the role of the *KNTC1* gene may vary by cancer type. In fact, mRNA expression data from The Human Protein Atlas revealed poor survival rates of patients with GC exhibiting a low expression of *KNTC1* (Data S1 and Fig. S3). Previous findings that the suppression of the expression of the *KNTC1* gene contributes to CIN may support these data (26-28). In the future, additional large-scale studies using clinical samples are warranted to clarify the association between the *KNTC1* gene and CIN and its causal association with patient prognosis.

In conclusion, the present study demonstrated that the knockdown of *KNTC1* increased the frequency of lagging chromosomes in GC cells. These finding suggest that the suppression of the expression of *KNTC1* may contribute to CIN in GC.

## Supplementary Material

Giemsa-stained image of the lagging chromosome in NCI-N87 cells. Chromosomes remained on the metaphase plate and formed bridges in anaphase (red arrows).

Cell proliferation following *KNTC1* knockdown. (A) No decrease in cell proliferation was observed in the MKN74, KATOIII and TIG-1-20 cells following *KNTC1* knockdown, whereas a decrease in cell proliferation was observed in the NCI-N87 cells. All data were analyzed using the Student's t-test. ^*^P<0.05 vs. siControl. (B) No increase in apoptosis was observed following *KNTC1* knockdown in all four cell lines. DAPI staining; 60X objective. *KNTC1*, kinetochore-associated 1 gene.

Kaplan-Meier plot showing *KNTC1* mRNA expression levels and outcomes of prognoses in patients with gastric cancer. In gastric cancer, a low *KNTC1* expression was found to be associated with a poor prognosis. P=0.047, low expression vs. high expression. Kaplan-Meier plots were obtained from The Human Protein Atlas version 23.0) (https://www.proteinatlas.org/ENSG00000184445-KNTC1/cancer/stomach+cancer#STAD_TCGA). *KNTC1*, kinetochore-associated 1 gene.

Supplementary materials and methods

## Data Availability

The data generated in the present study may be requested from the corresponding author.
