# Supplementary material for: Downregulation of kinetochore-associated 1 gene increases lagging chromosomes and contributes to chromosomal instability in gastric cancer cells
Source: Med Int (Lond). 2025 Aug 4;5(5):59. doi: 10.3892/mi.2025.258 (PMC12360148; doi:10.3892/mi.2025.258)

Figure S1. Giemsa-stained image of the lagging chromosome in NCI-N87 cells. Chromosomes remained on the metaphase plate and formed bridges in anaphase (red arrows).

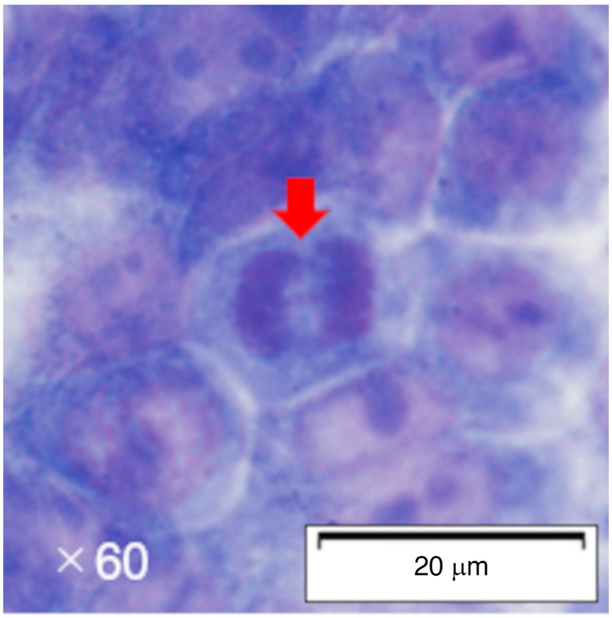

Figure S2. Cell proliferation following *KNTC1* knockdown. (A) No decrease in cell proliferation was observed in the MKN74, KATOIII and TIG-1-20 cells following *KNTC1* knockdown, whereas a decrease in cell proliferation was observed in the NCI-N87 cells. All data were analyzed using the Student's t-test. *P<0.05 vs. siControl. (B) No increase in apoptosis was observed following *KNTC1* knockdown in all four cell lines. DAPI staining; 60X objective. *KNTC1*, kinetochore-associated 1 gene.

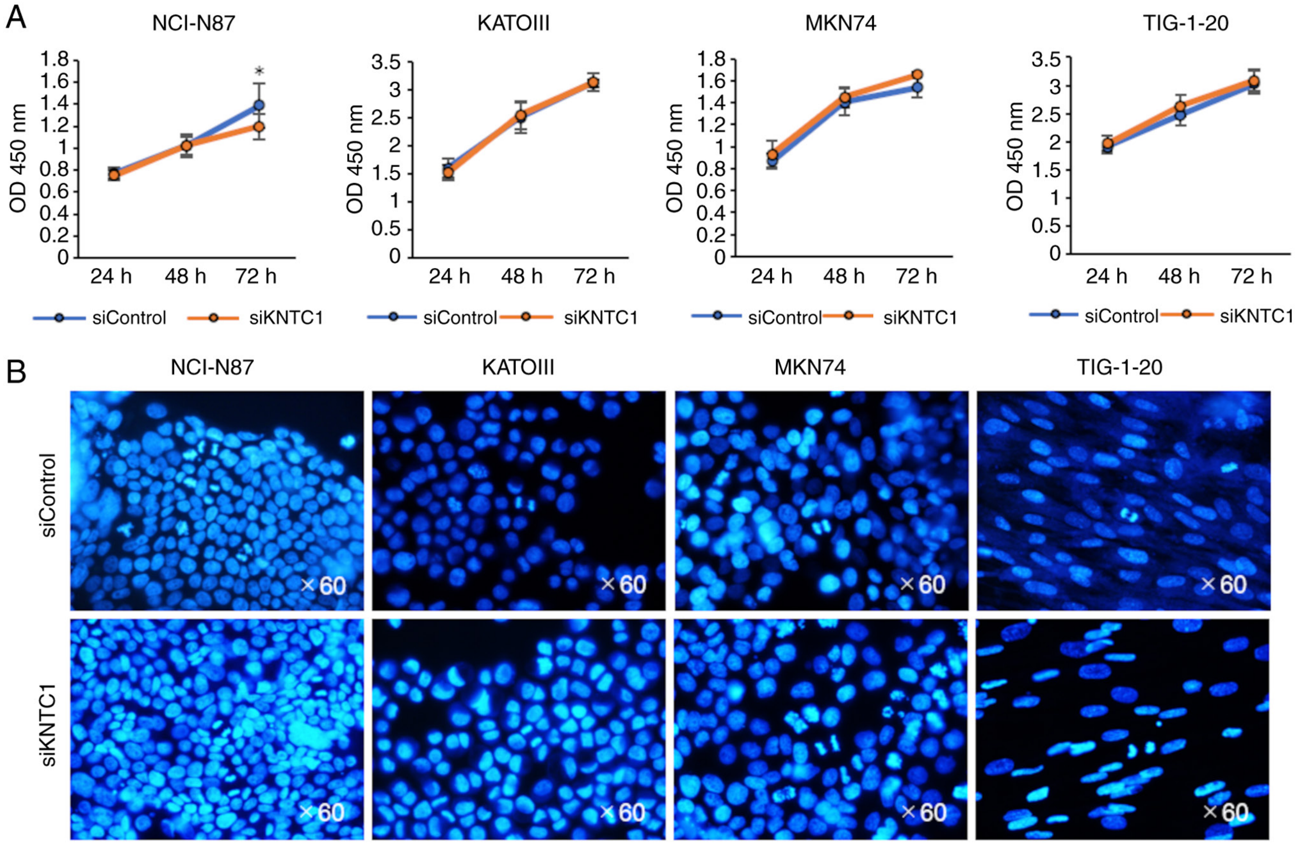

Figure S3. Kaplan-Meier plot showing *KNTC1* mRNA expression levels and outcomes of prognoses in patients with gastric cancer. In gastric cancer, a low *KNTC1* expression was found to be associated with a poor prognosis. P=0.047, low expression vs. high expression. Kaplan-Meier plots were obtained from The Human Protein Atlas version 23.0) (https://www.proteinatlas.org/ENSG00000184445- KNTC1/cancer/stomach+cancer#STAD_TCGA). *KNTC1*, kinetochore-associated 1 gene.

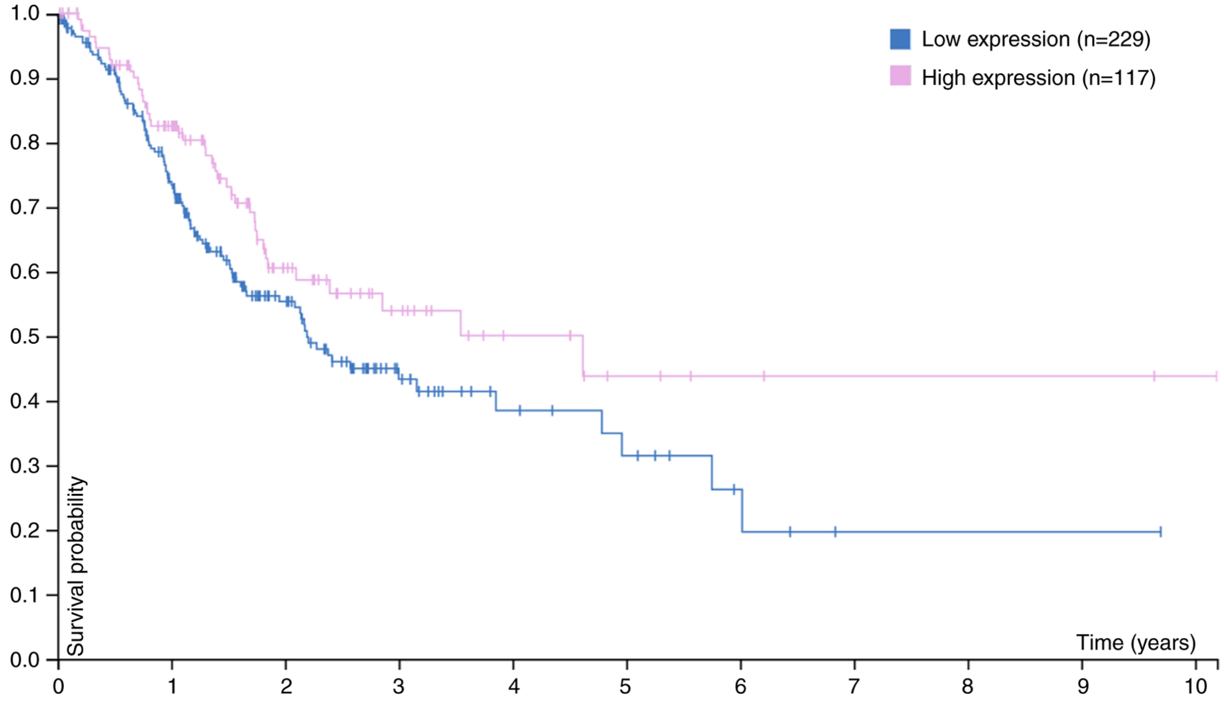

Supplement: Giemsa-stained image of the lagging chromosome in NCI-N87 cells. Chromosomes remained on the metaphase plate and formed bridges in anaphase (red arrows). [file Supplementary_Data1.pdf]
