# Supplementary material for: Downregulation of kinetochore-associated 1 gene increases lagging chromosomes and contributes to chromosomal instability in gastric cancer cells
Source: Med Int (Lond). 2025 Aug 4;5(5):59. doi: 10.3892/mi.2025.258 (PMC12360148; doi:10.3892/mi.2025.258)
Supplement: Supplementary materials and methods [file Supplementary_Data2.pdf]

Data S1: Supplementary materials and methods

Giemsa staining. The cells were fixed with methanol (cat. no. 131-01826; Wako Pure Chemical Corporation) for 2 min at room temperature. Following fixation, the slides were immersed in Giemsa staining solution (cat. no. 1.09204.0500; Merck KGaA) for 25 min at room temperature. Giemsa staining solution was diluted 1:20 with 1/150 M phosphate buffer (cat. no. 73115; Kanto Chemical Corporation) (pH 6.4). The slides were then rinsed with tap water for 20-30 sec and air dried. After drying, the slides were sealed and the lagging chromosomes were observed under a light microscope (BX53F; Olympus Corporation).

KNTC1 mRNA expression level and survival data of patients with gastric cancer. Kaplan-Meier plots summarizing the association between the kinetochore-associated 1 (*KNTC1*) mRNA expression levels and survival in patients with gastric cancer. Data were obtained from The Human Protein Atlas (https://www.proteinatlas.org/) (1).

Analysis of cell proliferation after KNTC1 knockdown. The NCI-N87, KATOIII, MKN74 and TIG-1-20 cells were seeded in 96-well culture plates at a density of $1.0 x 10^4$ cells/well and transfected with siRNA targeting *KNTC1* or negative control (siControl) after 24 h. At 1, 2 and 3 days following transfection, 10 μl reagent from a Cell Counting Kit-8 (cat. no. CK04; Dojindo Laboratories, Inc.) were added to each well, and the cells were cultured for 4 h at 37˚C. Cell proliferation was measured by assessing the absorption at 450 nm using a Trister2 LB942 Multimode Reader (Berthold Technologies).

Analysis of apoptotic bodies following KNTC1 knockdown. The NCI-N87, KATOIII, MKN74 and TIG-1-20 cells were seeded into 4-well culture slides and cultured for 24 h at 37˚C. After 24 h, the cells were transfected with siRNA targeting *KNTC1* or Negative Control (siControl). Following transfection, the cells are incubated for an additional 3 days and fixed for 20 min at room temperature with 4% paraformaldehyde. The slides were washed twice for 5 min each with phosphate-buffered saline and sealed using coverslips and VECTASHIELD Vibrance Antifade Mounting Medium with DAPI. Apoptotic bodies were observed fluorescence microscope (2).

References

1. Uhlén M, Fagerberg L, Hallström BM, Lindskog C, Oksvold P, Mardinoglu A, Sivertsson Å, Kampf C, Sjöstedt E, Asplund A, *et al*: Tissue-based map of the human proteome. Science 347: 1260419, 2015.
2. Wu RS, Wu KC, Yang JS, Chiou SM, Yu CS, Chang SJ, Chueh FS and Chung JG: Etomidate induces cytotoxic effects and gene expression in a murine leukemia macrophage cell line (RAW264.7). Anticancer Res 31: 2203-2208, 2011.
